# Transcriptome analysis and development of EST-SSR markers in the mushroom *Auricularia heimuer*

**DOI:** 10.1038/s41598-024-63080-1

**Published:** 2024-05-29

**Authors:** Lihe Jiao, Chuang Han, Jianan Zhu, Piqi Zhang, Yinpeng Ma, Xiaodong Dai, Yunzhi Zhang

**Affiliations:** Institute of Microbiology, Heilongjiang Academy of Sciences, Harbin, 150010 China

**Keywords:** *Auricularia heimuer*, Transcriptome, EST-SSRs, Genetic diversity, Population structure, Genetics, Microbiology

## Abstract

*Auricularia heimuer*, the third most frequently cultivated edible mushroom species worldwide, has high medicinal value. However, a shortage of molecular marker hinders the efficiency and accuracy of genetic breeding efforts for *A. heimuer*. High-throughput transcriptome sequencing data are essential for gene discovery and molecular markers development. This study aimed to clarify the distribution of SSR loci across the *A. heimuer* transcriptome and to develop highly informative EST-SSR markers. These tools can be used for phylogenetic analysis, functional gene mining, and molecular marker-assisted breeding of *A. heimuer*. This study used Illumina high-throughput sequencing technology to obtain *A. heimuer* transcriptome data. The results revealed 37,538 unigenes in the *A. heimuer* transcriptome. Of these unigenes, 24,777 (66.01%) were annotated via comparison with the COG, Pfam, and NR databases. Overall, 2510 SSRs were identified from the unigenes, including 6 types of SSRs. The most abundant type of repeats were trinucleotides (1425, 56.77%), followed by mononucleotides (391, 15.58%) and dinucleotides (456, 18.17%). Primer pairs for 102 SSR loci were randomly designed for validity confirmation and polymorphism identification; this process yielded 53 polymorphic EST-SSR markers. Finally, 13 pairs of highly polymorphic EST-SSR primers were used to analyze the genetic diversity and population structure of 52 wild *A. heimuer* germplasms, revealing that the 52 germplasms could be divided into three categories. These results indicated that SSR loci were abundant in types, numbers, and frequencies, providing a potential basis for germplasm resource identification, genetic diversity analysis, and molecular marker-assisted breeding of *A. heimuer*.

## Introduction

*Auricularia heimuer*, a wood-decaying mushroom, is the third most frequently cultivated edible mushroom worldwide^1^. Globally, China has the highest cultivated yield of *A. heimuer*, and Northeast China contributes approximately 70% of the national yield. *A. heimuer* is an important food source in Asia because of its high protein, trace element, vitamin, and carbohydrate contents, as well as its low fat content^2,3^. It also has high medicinal potential^4^, including anti-tumor, antioxidant, anticoagulant, and hypolipidemic properties^5–9^. Thus far, *A. heimuer* strains have mainly been derived from the domestication of wild strains and isolation of natural mutants. Decreases in wild resources and gradual convergence through artificial selection have led to reduced genetic diversity among *A. heimuer* strains^10^. Additionally, it is difficult to distinguish *A. heimuer* strains via morphological characteristics, hindering the dissemination of enhanced *A. heimuer* and the process of cultivar improvement. Thus, there is a need to establish and improve methods for analyzing genetic diversity in *A. heimuer*. Research priorities include effective evaluation of germplasm resources and establishment of a genetic database for the protection and scientific utilization of *A. heimuer* resources^11^.

In non-model plants without reference genomes, the use of molecular markers has become a key method for studies of diversity levels, population genetic structures, and genetic relationships of species germplasm resources^12^. This method is not affected by environmental stress, natural selection, or disease susceptibility^13^. Molecular markers are specific DNA fragments that directly represent genetic variation at the DNA level and can reveal specific genomic differences among individuals or populations^14^. In previous studies, molecular markers such as ISSR, SRAP, TRAP, and SCAR were successfully used to study *A. heimuer*^15–19^. A key molecular marker, the simple sequence repeat (SSR), is widely used for identification. This marker is easy to use, demonstrates good versatility and polymorphism properties, exhibits good stability, and can be developed in a simple manner^20,21^. SSR markers can be divided into two types based on the source: gSSR and EST-SSR^22^. gSSR is randomly distributed on the genome, and EST-SSR is located in the expression sequence, which is a molecular marker directly related to functional genes. Because ESTs are derived from coding DNA, their flanking sequences are usually highly conserved, allowing EST-SSR markers to achieve greater versatility^23^. This property supports the implementation of molecular marker technology in plant research. Additionally, due to their codominant and often single-locus nature, SSR loci can be recognized across different genotypes within the same species and frequently in the genotypes of closely related species. Thus, SSR loci can be efficiently transferred to related species^24,25^.

Advances in high-throughput sequencing have led to the development of EST-SSR markers, which offers new methods for assessment of species diversity in many edible mushrooms, such as *Lentinula edodes*, *Agaricus subrufescens*, *Bailinggu*, *Morchella esculenta*, and *A. heimuer*^26–30^. To enhance the protection and utilization of wild germplasm resources, EST-SSR markers based on transcriptome sequencing and genetic analysis of *A. heimuer* have been developed. In this study, Illumina high-throughput sequencing technology was used to obtain *A. heimuer* transcriptome data, which were then subjected to assembly and functional annotation. Subsequently, the frequencies, distributions, and functions of SSR markers in the *A. heimuer* transcriptome were analyzed. Next, EST-SSRs were established and polymorphism levels were detected. Finally, EST-SSRs were used to evaluate genetic diversity and genetic structures in wild *A. heimuer*.

## Results

### De novo assembly of the transcriptome

In total, 465,568,126 raw reads and 443,583,204 clean reads (95.28%) were generated (Table 1), with a Q20 base percentage of 98.89% and a GC percentage of 60.92%. Trinity software assembled high-quality reads into 147,073 contigs, with a mean length of 1066 bp and an N50 length of 1742 bp. These contigs were then spliced into 37,538 unigenes, with a total length of approximately 36.7 Mb, a mean length of 979.94 bp, and an N50 length of 1748 bp. There were 19,022 (50.67%) unigenes between 200 and 500 bp, 14,133 (37.65%) unigenes between 501 and 2000 bp, and 4383 (11.68%) unigenes longer than 2000 bp.

**Table 1:** Summary of the analysis of *A. heimuer* transcriptomic data.

Number of raw reads	465,568,126
Number of high-quality reads	443,583,204
Number of clean nucleotides (nt)	62,819,299,427
Total length of contigs (bp)	156,856,636
Total length of unigenes (bp)	36,785,052
Number of contigs	147,073
Number of unigenes	37,538
Total number of identified SSRs	2510
Number of SSR-containing sequences	2216
Number of sequences containing more than one EST-SSR	232

### Gene annotation based on different databases

**Figure 1:**
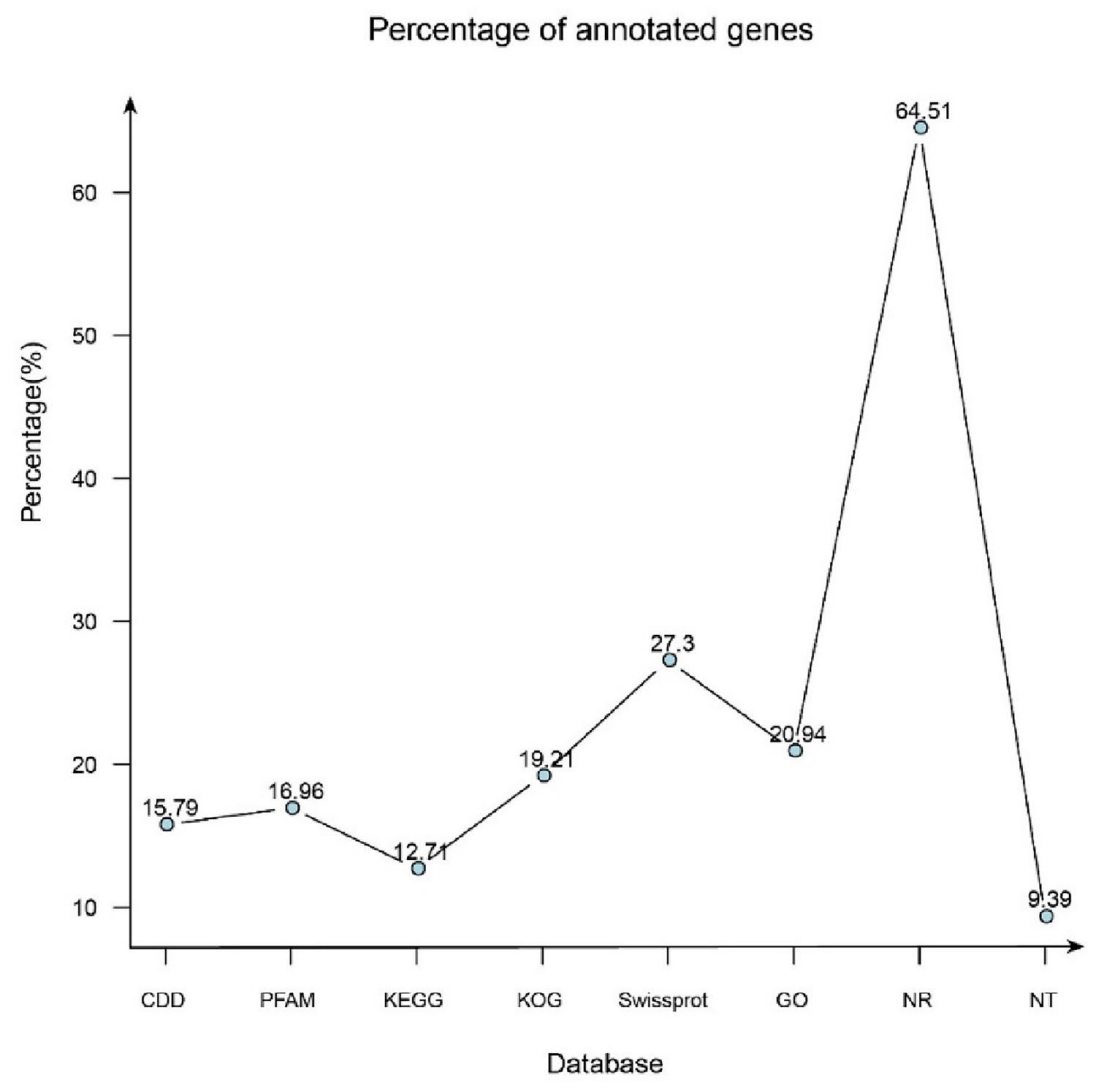
Frequency and distribution of SSRs on the basis of the motif.

**Figure 2:**
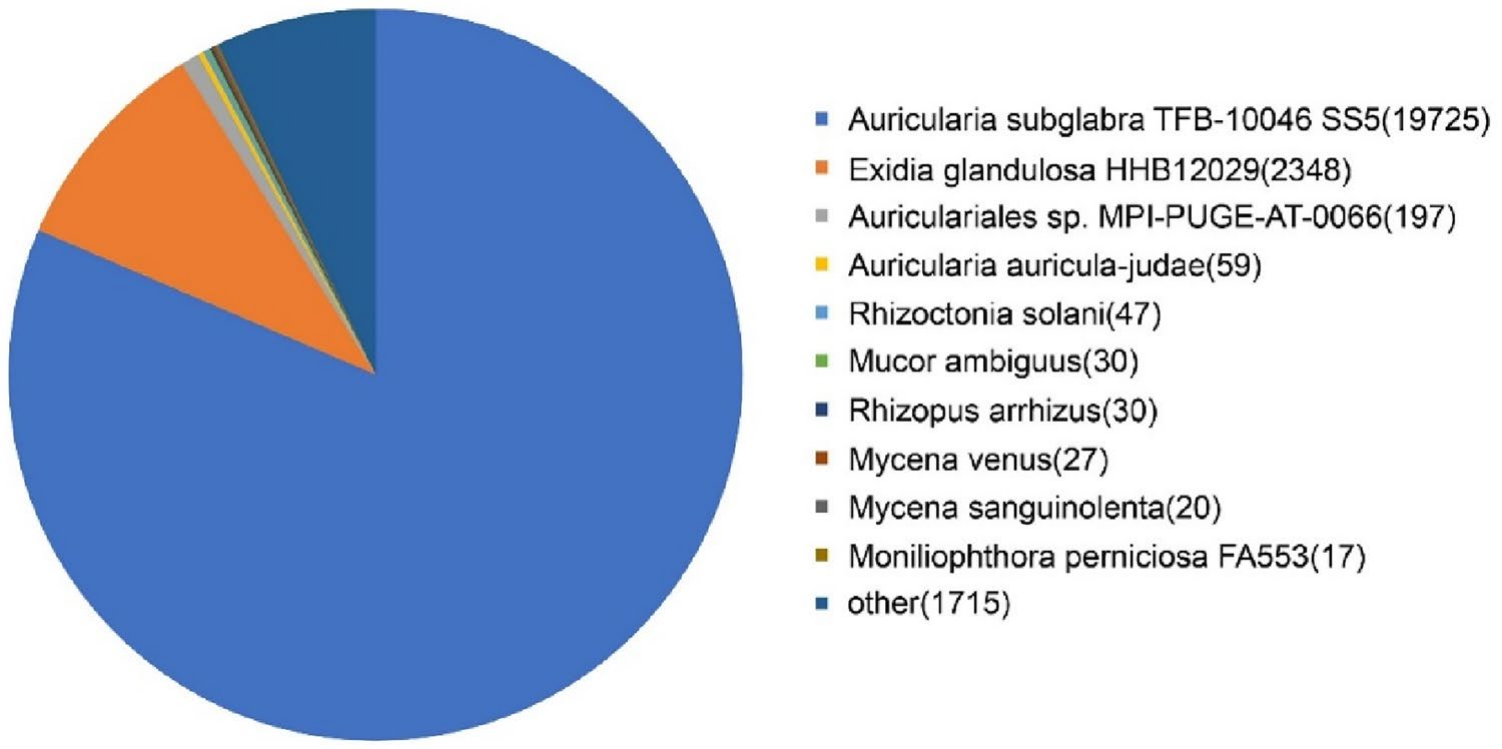
Homologous species distribution of *A. heimuer* transcripts annotated in the NR database.

Sequence similarity was determined by BLAST algorithm. The functions of unigene sequences were evaluated and classified using the conserved domain database (CDD), Pfam, Kyoto encyclopedia of genes and genomes (KEGG), and KOG databases, as well as other databases. Because of the different databases used, the number of functional annotations greatly varied among the unigenes. The NR database had the highest number of annotated unigenes (24,215, 64.51%), whereas the NT database had the lowest number of annotated unigenes (3,525, 9.39%) (Fig. 1). According to the species distribution annotation, *Auricularia subglabra* had the highest percentage of unigenes (52.55%). This was followed by *Exidia glandulosa*, *Auriculariales* sp., *Auricularia auricula-judae*, *Rhizoctonia solani*, *Mucor ambiguus*, *Rhizopus arrhizus*, *Mycena venus*, *Mycena sanguinolenta*, and *Moniliophthora perniciosa* with percentages of 6.25%, 0.52%, 0.16%, 0.13%, 0.08%, 0.08%, 0.07%, 0.05%, and 0.05%, as indicated in Fig. 2.

**Figure 3:**
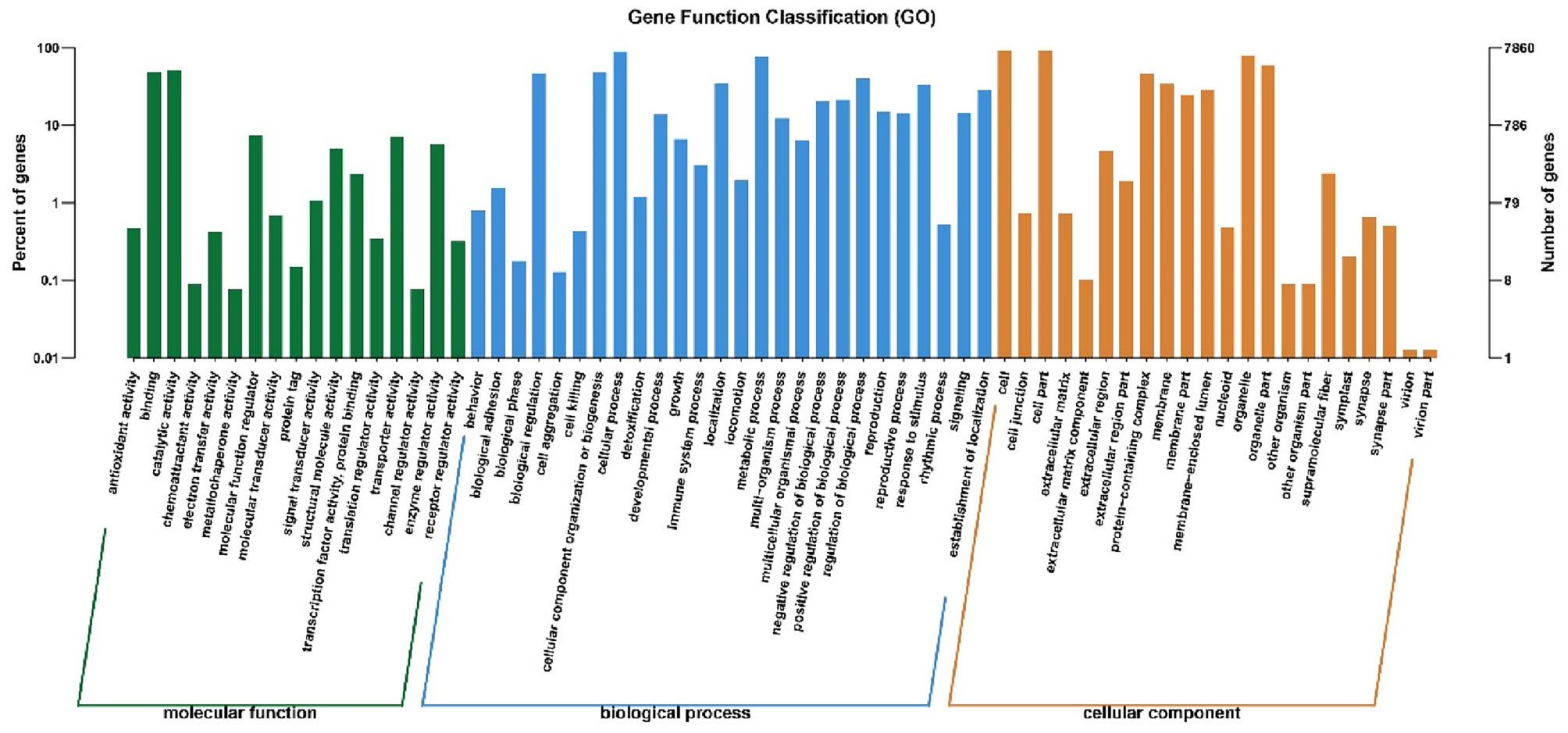
GO classification of assembled SSR-containing unigenes in *A. heimuer*.

The functions of the identified genes were explored by graphene oxide analysis. According to the degree of graphene oxide enrichment, 7860 unigenes from *A. heimuer* were divided into 65 functional groups and assigned to three main categories: biological process (BP; 41,851, 46.95%), cellular component (CC; 36,996, 41.50%), and molecular function (MF; 10,292, 11.55%) (Fig. 3). The 65 subcategories were then subdivided into Gene Ontology terms. The three main BP subcategories were cellular process (7046, 16.84%), metabolic process (6030, 14.41%) and cellular component organization or biogenesis (3760, 8.98%). The three main CC subcategories were cell (7270, 19.65%), cell part (7270, 19.65%), and organelle (6294, 17.01%). Finally, the three main MF subcategories were catalytic activity (4035, 39.20%), binding (3801, 36.93%), and molecular function regulator (581, 5.65%).

KEGG analysis revealed that 12,551 (33.44%) of the 37,538 unigenes could be classified into 279 pathways. The most representative metabolic pathways were metabolism (3209, 25.57%), followed by genetic information processing (1775, 14.14%), organismal systems (1,405, 11.19%), cellular processes (1197, 9.54%), and environmental information processing (858, 6.84%). Functional analysis showed that unigenes were mainly enriched in the biosynthesis of amino acids (240) and RNA transport (240), as shown in Table S3.

**Figure 4:**
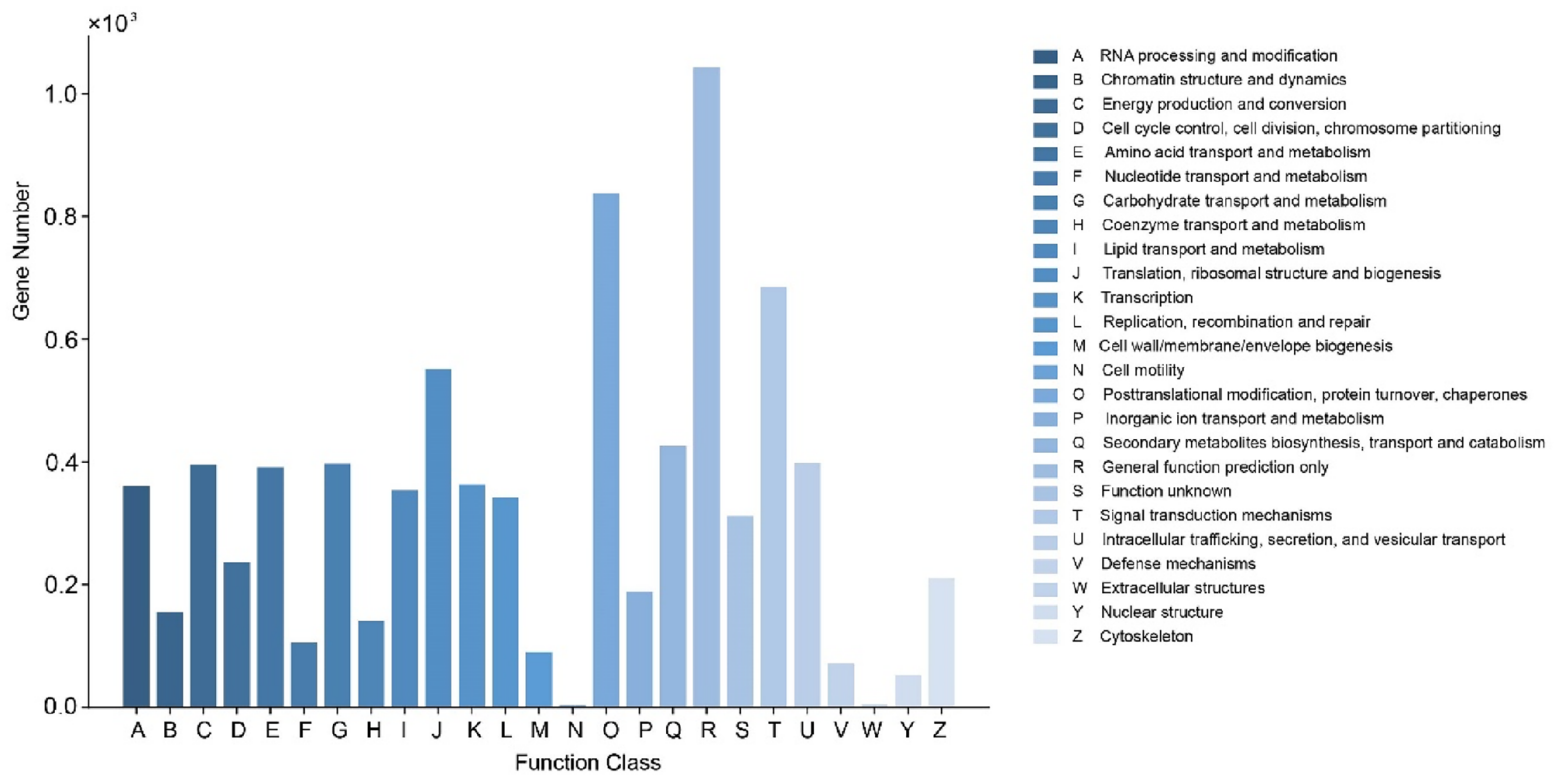
KOG classification of the SSR-containing unigenes.

The KOG database was used to compare all unigenes from *A. heimuer*, with the goal of predicting the functions of *A. heimuer* genes. Upon annotation by KOG, 8921 unigenes could be classified into 25 categories. The largest predicted category was general function (1041, 11.67%). This was followed by posttranslational modification, protein turnover, and chaperones (836, 9.37%); signal transduction mechanisms (684, 7.67%); and translation, ribosomal structure, and biogenesis (550, 6.17%). The smallest predicted categories were cell mobility (2, 0.02%) and extracellular structures (4, 0.04%) (Fig. 4).

### Frequency and distribution of SSRs

SSR markers play important roles in genetic diversity analysis, genetic mapping, and molecular marker-assisted breeding because of their wide distribution, co-dominance, high repeatability, large number of polymorphisms, and easy operation^31^. In total, 2510 EST-SSR loci were identified in 37,538 unigenes; among these unigenes, 232 had two or more SSR loci. The most common motifs were single repeats (391, 15.58%), double repeats (456, 18.17%) and triple repeats (1425, 56.77%).

**Figure 5:**
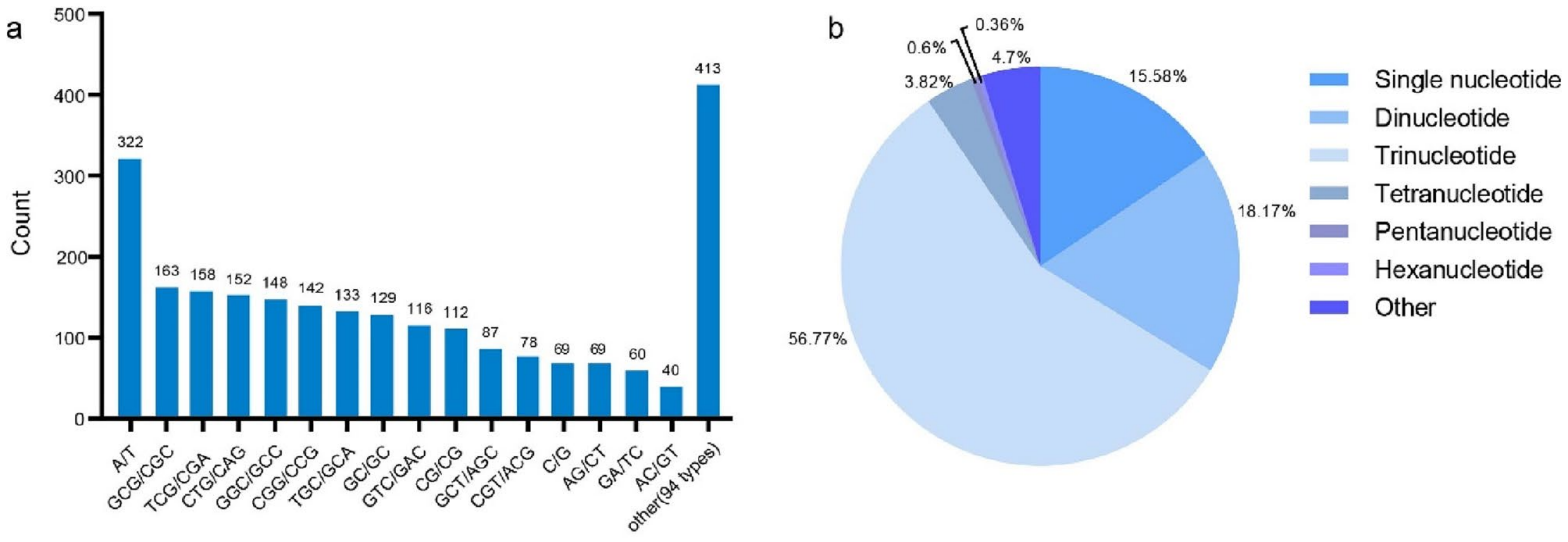
The SSRs repeated nucleotide types for *A. heimuer*.

Among all SSR markers, most were trinucleotide repeats (1425, 56.77%); the next most common markers were dinucleotide repeats (456, 18.17%). The numbers of mononucleotide repeats, tetranucleotide repeats, pentanucleotide repeats, and hexanucleotide repeats were 391, 96, 15, and 9, respectively, comprising 15.58%, 3.82%, 0.60%, and 0.36% of all markers (Fig. 5b). A/T was the most common single-base repeat (82.35%), GC/GC was the most common two-base repeat (28.29%), and GCG/CGC was the most common three-base repeat (11.44%); TACC/GGTA, and AACCA/TGGTT were the most common four-base and five-base repeats, respectively (Fig. 5a).

### Genetic diversity and structure analysis

**Figure 6:**
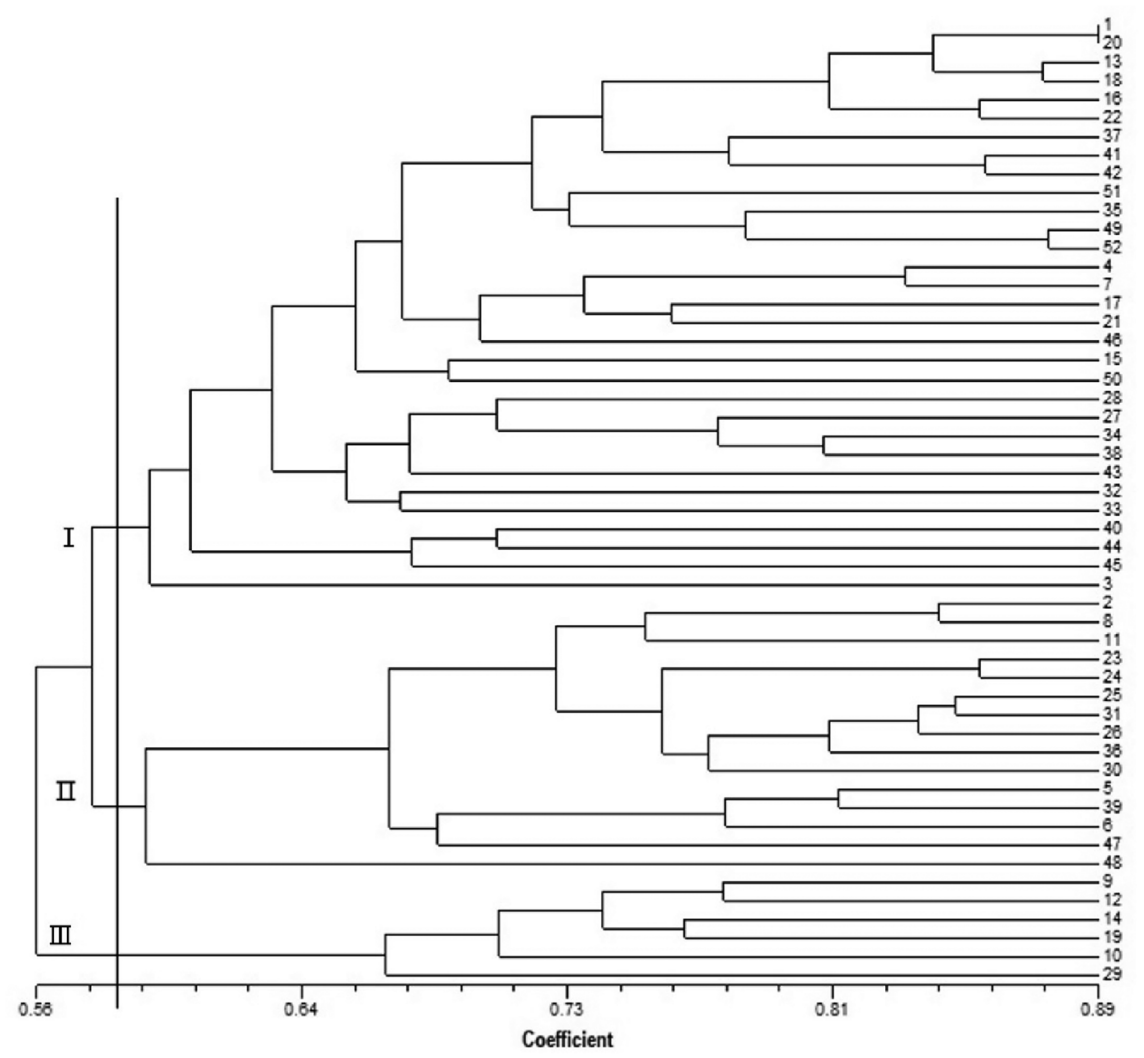
The UPGMA analysis among 52 wild *A. heimuer*.

Of the 102 primer pairs, 97 were able to amplify PCR products from genomic DNA, and 43 PCR reactions produced a single band. Finally, 13 primer pairs of highly polymorphic EST-SSR primers were used to analyze the genetic diversity and population structure of 52 wild *A. heimuer* germplasms. The genetic similarity coefficients of 52 strains of *A. heimuer* ranged from 0.56 to 0.89, according to a cluster map of SSR markers constructed by NTSys using the unweighted pair group method with arithmetic mean (UPGMA) approach. Using a similarity index threshold of 0.58, 52 samples were divided into three main groups (Fig. 6). Class I contained 31 samples; of these, strains with a genetic similarity coefficient > 0.85 comprised 32.26%. Strains HMCC50008 and HMCC50931 were closely related, with a similarity coefficient of 0.89. Among the 15 samples in group II, HMCC50116 was genetically distant from other strains (similarity coefficient of 0.59). Class III contained 6 samples, with a similarity coefficient that ranged from 0.66 to 0.77.

**Figure 7:**
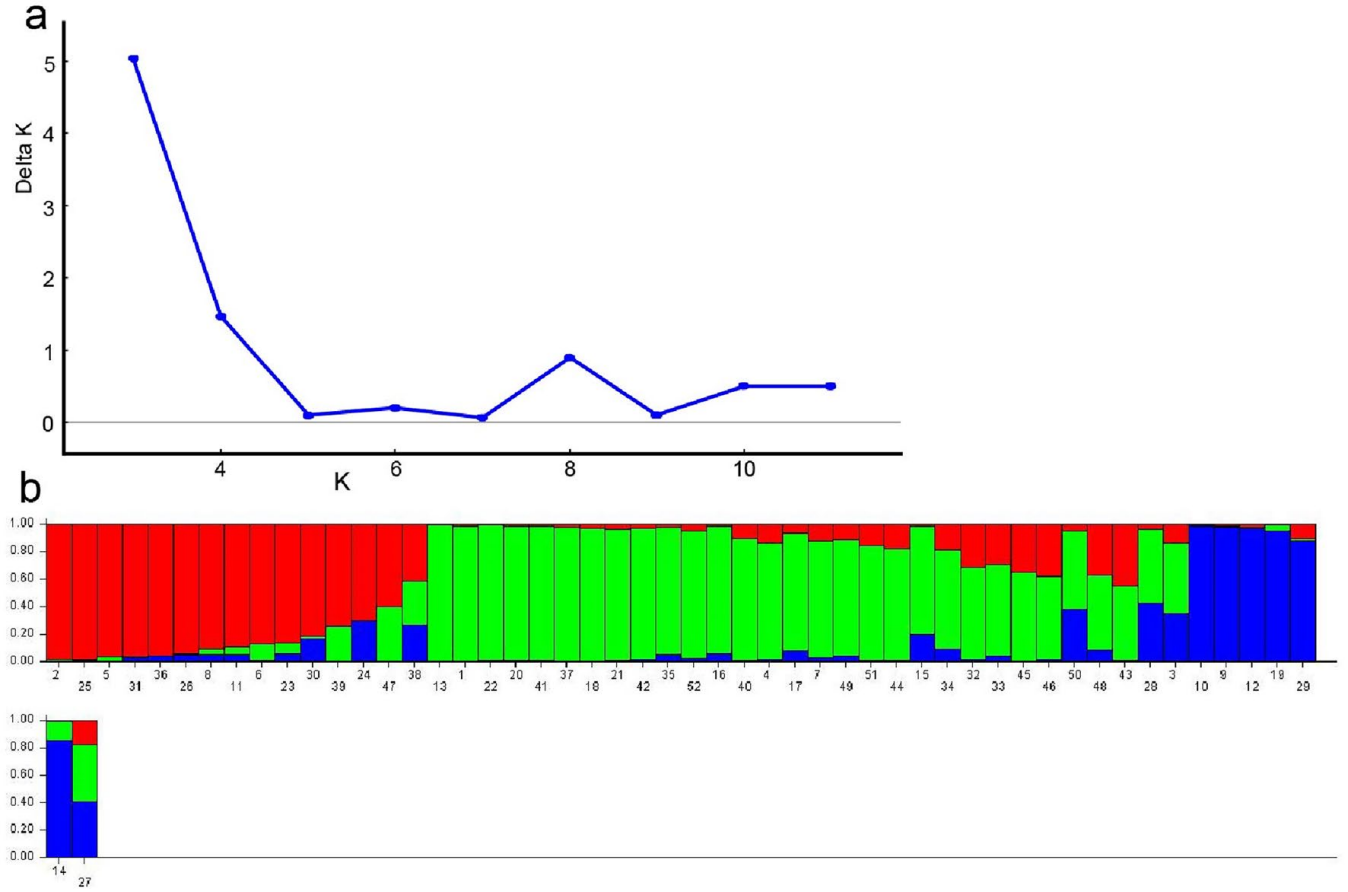
Results of STRUCTURE analysis for 52 populations using 13 EST-SSR markers. (**a**) Estimation of population using *ΔK* value withcluster *K* ranging from 2 to 12. (**b**) Estimation of population structure based on STRUCTURE analysis.

Vertical bars with various colors represent sample membership coefficients estimated using the Q statistic. The graph shows the membership scores of the *ΔK* estimator derived using LnP (D), with *K* ranging from 1 to 10. The number of *A. heimuer* strains cultivated in Northeast China was assumed to range from 2 to 12, and the calculation was repeated 15 times. Trend analysis of *ΔK* showed that when *delt* was 3, the *ΔK* value reached an obvious peak (Fig. 7a); all samples were then divided into the following three groups (Fig. 7b). Group I (red) comprised 15 samples, group II (green) comprised 31 samples, and group III (blue) comprised 6 samples. The UPGMA dendrogram shows that the germplasms can be divided into three groups (Fig. 6). Clustering analysis of the 52 wild *A. heimuer* strains revealed results that were largely consistent with the findings of population genetic structure analysis.

## Discussion

Advances in high-throughput sequencing technology and reduced sequencing costs have enhanced the efficiency and convenience of using transcriptomics data to identify SSR loci with large numbers of polymorphisms^32^. Compared with genomic-SSR molecular markers, EST-SSR molecular markers have lower development costs, greater versatility and conservation, and favorable associations with phenotypic traits^33^. In the present study, 2,510 SSR loci were identified from 37,538 unigenes in the *A. heimuer* transcriptome; the SSR frequency of 6.69% was higher than the rates for edible mushrooms such as *Auricularia polytricha* (4.70%) and *Pleurotus eryngii* (3.09%)^34,35^, indicating that the *A. heimuer* transcriptome contains abundant SSR loci. Factors such as database validity, species differences, and SSR search criteria may lead to differences in SSR frequency between *A. heimuer* and other analyzed species^36,37^.

SSR repeat types differ among species, but the dinucleotide and trinucleotide repeat types are dominant^38^. In the present study, SSRs were abundant in the *A. heimuer* transcriptome; trinucleotide repeats were most common (1425, 56.77%), followed by dinucleotide repeats (456, 18.17%). These results were consistent with previous findings regarding *Commelina communis*, *Phyllostachys violascens*, and Crataegus^39–41^. This study showed that differences in SSR number were partially related to the degree of species evolution; a high percentage of short repeat units indicated that the degree of species evolution was high^42^, implying that *A. heimuer* has a long evolutionary history and may have accumulated additional genetic variations. There is evidence that when the SSR length exceeds 20 bp, the polymorphism rate is high; a length of 12–19 bp is associated with a moderate polymorphism rate; a length below 12 bp leads to a low polymorphism rate^43^. In this study, 501 SSRs (19.96%) exceeded 20 bp in length.

SSR polymorphisms can be expressed as the difference in fragment length according to the numbers of base repeats and individual bases. Generally, as the number of repeats increases for a single base or a small number of bases, microsatellite sequences exhibit greater variability and increased polymorphism potential. In the present study, as the sequence copy number increased, the number of units of each SSR type in the *A. heimuer* genome tended to decrease. This phenomenon may be explained as follows: as the number of units of a particular SSR type increases, the SSR length also increases, resulting in outcomes such as greater instability and higher mutation rate; the long-term result is a decrease in the number of units of the affected SSR type^44^.

This study annotated the functions of unigenes containing SSRs; the functions of these potential SSR gene sequences mainly focused on biological activities such as metabolic processes, cellular component organization or biogenesis, catalytic activities, and regulators of molecular function. These biological activities are performed throughout plant growth and development, indicating that unigenes with SSR loci may have diverse gene functions. Additionally, this study demonstrated the utility of 13 newly developed polymorphic EST-SSR markers in evaluating genetic diversity among 52 wild *A. heimuer* germplasms in Northeast China. Among 102 primer pairs randomly selected for validation, 97 yielded distinct PCR product bands; this PCR success rate was higher than the rates for *Morchella spp.* and *Pleurotus geesteranus*^45,46^. In the analysis, the 13 markers divided the tested strains into three main groups according to UPGMA cluster analysis. The dendrogram revealed that wild *A. heimuer* individuals in Northeast China are highly diverse. The results of this study provide a scientific basis for future work regarding EST-SSR molecular markers, facilitating the use of functional genes and exploration of molecular marker-assisted breeding in *A. heimuer*.

## Conclusions

In this study, 2510 SSR loci were identified based on 37,538 unigenes obtained from the *A. heimuer* transcriptome. Furthermore, 13 EST-SSR markers were used to analyze the genetic diversity of 52 wild *A. heimuer* individuals. Our results showed that the populations of wild *A. heimuer* in Northeast China had a high level of genetic diversity. These findings provide molecular evidence to support molecular breeding, germplasm resource conservation, and core germplasm collection involving *A. heimuer*.

## Methods

### *A. heimuer* materials

Fifty-two wild *A. heimuer* strains were used in this study; all strains were provided by the Heilongjiang Provincial Microbial Germplasm Resources Preservation Center (National Edible Mushroom Germplasm Resources Library (Heilongjiang)). These strains had been collected from Northeast China for the development and characterization of EST-SSRs. Detailed sample information is provided in Supplementary Table S1.

### RNA extraction and transcriptome sequencing

The mycelia of *A. heimuer* were frozen and sent to Sangon Biotech (Shanghai) Co., Ltd. for extraction of total RNA. For each sample, 100 mg of *A. heimuer* mycelium were briefly washed in deionized water and frozen in liquid nitrogen. Total RNA was extracted from mycelium using Total RNA Extractor (TRIzol) (Sangon Biotech, Shanghai, China). RNA quality was verified using a Qubit 2.0 fluorometer (Thermo Fisher Scientific, Wilmington, DE, USA); RNA integrity and genomic contamination were assessed by RNase-free agarose gel electrophoresis. cDNA libraries were constructed. A normalized cDNA library of *A. heimuer* was constructed using methods described by Venugopal et al.^47–50^. Then, sequencing with an Illumina NovaSeq 6000 was performed to generate raw data. These raw Illumina sequencing data were submitted to the NCBI Sequence Read Archive (accession number, PRJNA1056119; http://www.ncbi.nlm.nih.gov/Traces/sra). The raw data underwent a series of quality assessments to obtain clean data. Transcripts were obtained by de novo assembly of clean data using the Trinity platform (https://github.com/trinityrnaseq/trinityrnaseq/wiki)^51^. The resulting transcripts were subjected to deduplication, and the longest transcript in each gene was regarded as a unigene; the set of unigenes was used for subsequent analyses.

### Functional classification notes

A transcriptome database was established after transcript splicing had been completed; next, comprehensive analysis and annotation of *A. heimuer* were performed. The non-redundant transcription sequences obtained via splicing were compared with seven databases (including the NCBI NR database and the EuKaryotic Orthologous Groups [KOG] database), yielding annotation data for wild *A. heimuer* unigenes. Comprehensive functional information was obtained for all transcripts, and annotations from each database were counted.

### EST-SSR mining and primer design

MISA (https://webblast.ipk-gatersleben.de/misa/)was used to search the SSR loci of the obtained unigenes. SSRs with < 6 single nucleotide repeats; < 6 dinucleotide repeats; and < 5 trinucleotide, tetranucleotide, pentanucleotide, and hexanucleotide repeats were filtered out. The sequence length on both sides of each SSR locus was > 100 bp; screening was conducted to identify compound SSRs with a base interval ≤ 100 bp. Primers were designed using Primer 3 (https://sourceforge.net/projects/primer3/)^52^, and 13 pairs of primers (synthesized by Shanghai Biotechnology Co., Ltd.; Shanghai, China) were randomly selected for polymerase chain reaction (PCR) amplification. The parameters for primer design were as follows: primer length, 18–25 bp; PCR product size, 100–300 bp; annealing temperature, 50–60 °C; and GC content, 40–60%. The specific sequences of primers used in this study are shown in Table S2.

### EST-SSR analysis

Wet mycelia from each strain were used for genomic DNA isolation. DNA was extracted and purified using the DNAsafe Plant Kit (TIANGEN, China). Each DNA sample was diluted to a working concentration of 50 ng/μL, and whole-genome DNA was stored at − 20 °C. In total, 102 primer pairs were randomly chosen and synthesized by Shanghai Biotechnology Co., Ltd. to develop polymorphic EST-SSR markers. After screening, 13 pairs of polymorphic primers were selected for analysis of genetic diversity in 52 wild *A. heimuer* strains. The PCR reaction system (20 μL) contained 10 μL of 2 × Rapid Taq Master Mix (Vazyme Biotech, Nanjing, China), 1 μL of primer, 1 μL of genomic DNA (50 ng), and ddH2O to a total volume of 20 μL. The PCR amplification conditions were hot start for 5 min at 94 °C, followed by 35 cycles of denaturation for 30 s 94 °C, annealing for 45 s at a primer-specific temperature, and extension for 1 min at 72 °C; the final step comprised extension for 10 min at 72 °C. PCR products were analyzed using an Amersham Imager 600 PCR gel imager (GE Healthcare, USA). PCR product bands on gel images observed were scored as present (1) or absent (0). Dice genetic similarity coefficient values were calculated using NTSYSpc (https://www.appliedbiostat.com/ntsyspc/update_NTSYSpc.html), then compared via the unweighted pair group method with arithmetic mean (UPGMA) for cluster analysis and system tree construction^40^. STRUCTURE software (version 2.3.4) was used to analyze the population structure of 52 *A. heimuer* individuals. Fifteen independent runs were performed for K values ranging from 2 to 12, each with 10,000 burn-in iterations and 500,000 Markov chain Monte Carlo repetitions. The online tool STRUCTURE HARVESTER was used to calculate the *ΔK* value indicating optimal population stratification. Graphs were plotted according to the optimal K value results^53,54^.

### Supplementary Information

Supplementary Tables.

## Data Availability

The data that support the findings of this study are available from the corresponding author upon reasonable request. The dataset is available from the NCBI BioProject (PRJNA1056119) and NCBI Short Read Archive (SRA) with accession number SRR27341122–SRR27341131.

## Abbreviations

SSR: Simple sequence repeats
gSSR: Genomic-SSR
EST-SSR: Expressed sequence tags-SSR
EST: Expressed sequence tag
Unigene: Universal gene
NR: Non-redundant protein
KOG: Eukaryotic homologous group
MISA: MIcroSAtellite Identification Tool
CDD: Conserved domains database
Pfam: The protein family database
KEGG: Kyoto encyclopedia of genes and genomes
KOG: Eu*K*aryotic Orthologous Groups
BP: Biological processes
CC: Cellular components
MF: Molecular functions
