# Supplementary material for: Transcriptome analysis and development of EST-SSR markers in the mushroom Auricularia heimuer
Source: Sci Rep. 2024 May 29;14:12340. doi: 10.1038/s41598-024-63080-1 (PMC11136984; doi:10.1038/s41598-024-63080-1)
Supplement: Supplementary file 1 — Supplementary Tables. [file 41598_2024_63080_MOESM1_ESM.docx]

**Table legends**

**Table S1. Wild *A.heimuer* germplasm accessions used in this study.**

**Table S2. Sequences of 102 primer pairs for EST-SSR markers.**

**Table S3. Summary of the unigenes mapped to KEGG functional pathways.**

**Table S1**

| NO. | Strain NO. | Origin | NO. | Strain NO. | Origin | NO. | Strain NO. | Origin |
| --- | --- | --- | --- | --- | --- | --- | --- | --- |
| 1 | HMCC50008 | Heilongjiang, China | 19 | HMCC50922 | Harbin, Heilongjiang, China | 37 | HMCC50097 | Harbin, Heilongjiang, China |
| 2 | HMCC50012 | Daxinganling, Heilongjiang, China | 20 | HMCC50931 | Daxinganling, Heilongjiang, China | 38 | HMCC50105 | Ordos, Inner Mongolia, China |
| 3 | HMCC50015 | Jixi, Heilongjiang, China | 21 | HMCC50936 | Yichun, Heilongjiang, China | 39 | HMCC50111 | Harbin, Heilongjiang, China |
| 4 | HMCC50017 | Daxinganling, Heilongjiang, China | 22 | HMCC51050 | Yanbian, Jilin, China | 40 | HMCC50114 | Harbin, Heilongjiang, China |
| 5 | HMCC50020 | Liaoning, China | 23 | HMCC50001 | Harbin, Heilongjiang, China | 41 | HMCC50118 | Yanbian, Jilin, China |
| 6 | HMCC50026 | Ordos, Inner Mongolia, China | 24 | HMCC50019 | Harbin, Heilongjiang, China | 42 | HMCC50871 | Shuangyashan, Heilongjiang, China |
| 7 | HMCC50028 | Yichun, Heilongjiang, China | 25 | HMCC50023 | Heihe, Heilongjiang, China | 43 | HMCC50934 | Harbin, Heilongjiang, China |
| 8 | HMCC50031 | Mudanjiang, Heilongjiang, China | 26 | HMCC50024 | Yichun, Heilongjiang, China | 44 | HMCC52212 | Jilin, China |
| 9 | HMCC50042 | Heihe, Heilongjiang, China | 27 | HMCC50033 | Daxinganling, Heilongjiang, China | 45 | HMCC50018 | Heihe, Heilongjiang, China |
| 10 | HMCC50046 | Russia | 28 | HMCC50040 | Yichun, Heilongjiang, China | 46 | HMCC50104 | Harbin, Heilongjiang, China |
| 11 | HMCC50048 | Mudanjiang, Heilongjiang, China | 29 | HMCC50044 | Mudanjiang, Heilongjiang, China | 47 | HMCC50106 | Ordos, Inner Mongolia, China |
| 12 | HMCC50055 | Inner Mongolia, China | 30 | HMCC50050 | Yichun, Heilongjiang, China | 48 | HMCC50116 | Mudanjiang, Heilongjiang, China |
| 13 | HMCC50066 | Jixi, Heilongjiang, China | 31 | HMCC50064 | Jixi, Heilongjiang, China | 49 | HMCC50192 | Jilin, China |
| 14 | HMCC50075 | Heihe, Heilongjiang, China | 32 | HMCC50070 | Daxinganling, Heilongjiang, China | 50 | HMCC50863 | Daxinganling, Heilongjiang, China |
| 15 | HMCC50081 | Jilin, China | 33 | HMCC50073 | Daxinganling, Heilongjiang, China | 51 | HMCC50875 | Yichun, Heilongjiang, China |
| 16 | HMCC50086 | Daxinganling, Heilongjiang, China | 34 | HMCC50078 | Jixi, Heilongjiang, China | 52 | HMCC50879 | Harbin, Heilongjiang, China |
| 17 | HMCC50093 | Mudanjiang, Heilongjiang, China | 35 | HMCC50085 | Heihe, Heilongjiang, China |  |  |  |
| 18 | HMCC50141 | Mudanjiang, Heilongjiang, China | 36 | HMCC50096 | Daxinganling, Heilongjiang, China |  |  |  |

**Table S2**

| Prime No. | SSR | FORWARD PRIMER1 (5'-3') | Prime No. | SSR | FORWARD PRIMER1 (5'-3') |
| --- | --- | --- | --- | --- | --- |
| E1-F | (CGC)5 | ACCATACGACCTTGTCCTGC | E52-F | (CA)7 | GGTTCACGTACAACCCCAAC |
| E1-R |  | TTCCAGAGCGCCGTAAGTAT | E52-R |  | GCAAATGAGCGAAAGGAAAG |
| E2-F | (GCT)5 | ATCCTTCGACTTTCACGCAC | E53-F | (TG)6 | TCTGGCTTTGGCTTCACTTT |
| E2-R |  | CAAGACCCTGCCTATTGCTC | E53-R |  | CTCAGGTCCAGCATACGACA |
| E3-F | (CAG)5 | CTGATATGGGGTTTGGATGG | E54-F | (CG)6 | CGCCTTCTTTTTCCTGACTG |
| E3-R |  | CATAACCTTTGCTTTCCCCA | E54-R |  | CATCATTTGCCACTTGCATC |
| E4-F | (GCT)5 | GCTGCTCCTTCCTTCTCCTT | E55-F | (GC)6 | GGTTCTGGTTGTGGTGCTG |
| E4-R |  | ACATTTTATTGCCTGGCTGG | E55-R |  | CACCACACCGTCTTCATTTG |
| E5-F | (TCG)5 | GTCCTCGACGTCCATCTTGT | E56-F | (CG)6 | AGTCAGTCGGGGAGCAACT |
| E5-R |  | CTTTAGCAAGGGTAAGCCCA | E56-R |  | ATCTGGTCCGAGATCGTGTC |
| E6-F | (AAG)5 | CGACCGGTTTGACTTTTCAT | E57-F | (CT)7 | TTTCTTTGTTTCCCGACGAC |
| E6-R |  | CTGGGCATCACCTTCTTCAT | E57-R |  | TTTGAAGAACCCCGCATAAC |
| E7-F | (GGA)5 | CCTCGCAGCCATCTTTCTAC | E58-F | (GC)6 | GACGACCTCCTTCTCCACC |
| E7-R |  | GGAGATGCAGGCAAAGGATA | E58-R |  | TTAAGGACCGGTGGAATCTG |
| E8-F | (GAC)5 | GAAGTCGACGAGGAGAAACG | E59-F | (GC)7 | GGAGGAAGATGAGGAGGAGG |
| E8-R |  | CCTTATCGTTGGTGGTGAGG | E59-R |  | CCTCCTCGAGCAGAGCATT |
| E9-F | (CAG)7 | AACCAGCTGCGGTCATACTT | E60-F | (GC)6 | AGACTCGAATCTGACGCGAT |
| E9-R |  | GGTATCATGACCATCCCGAC | E60-R |  | ACTACACCGCGGTAGACGAC |
| E10-F | (GCC)5 | GAGTTGATTTGGCCGTGTTT | E61-F | (CT)6 | CAAAGACGCATTACGACCCT |
| E10-R |  | CGGTAGCAGTAAAAGCGGAG | E61-R |  | CAACGCGAGTGATCAAGAAA |
| E11-F | (CAG)5 | GGACTCGTACAACTCGAGCC | E62-F | (GA)6 | AGCCTTCCTCCCTCTCTCAG |
| E11-R |  | AGAGCTGCGTAGCAGAGAGC | E62-R |  | AATAAACCATCTGGCTGGCA |
| E12-F | (CGC)8 | CGTGCATAAACTGTAGGCGA | E63-F | (GT)6 | CATGCGAGTTACTCCACGAA |
| E12-R |  | CTTCGAGTGCGTGTGTTGTC | E63-R |  | CCCTCGAGTGGAAAGTTTGT |
| E13-F | (GCA)5 | AAGCAGAAGATGGGCAAGTG | E64-F | (CG)6 | CCCCTCCACAGCCTCTATTC |
| E13-R |  | TGGTCGAACGGGTAGAAGAG | E64-R |  | GAATCTGCTTCCACAGGAGC |
| E14-F | (GCA)6 | CAATAATGGCACTGCAGCC | E65-F | (GT)6 | CTGATAATGAAAGCAGCGCA |
| E14-R |  | CTGCTATTACTCCTGCCGCT | E65-R |  | ATGTTTCTGCTCTGGATCGG |
| E15-F | (GGA)7 | GAGAAGGAATGCGGAGAATG | E66-F | (GC)6 | GATCGAGGAGGACGAGGAC |
| E15-R |  | AGCGAGAAGGTGAGCGTG | E66-R |  | AGCAGTTGAAGCAACAGCAA |
| E16-F | (CCG)8 | GGGAAACAAGGGTGTAGGGT | E67-F | (GC)6 | TTATTCTTTGGTTGGGCGAG |
| E16-R |  | AGCTCGAACAAGGACGAGG | E67-R |  | CCCCAGTATGTCACGAGTCC |
| E17-F | (GAC)6 | TCGGTCGAAGTCTTCAAAGC | E68-F | (CT)7 | ATGTATGAGTTCGGGCAACC |
| E17-R |  | GACGCAGCAATATGAAAGCA | E68-R |  | TACGTTGCCCATGAATACCA |
| E18-F | (CCG)5 | TATGCCTCGTTGAGCTTGTG | E69-F | (GGTT)5 | TATTGGGCTGTGGTAGGGAG |
| E18-R |  | CGAGTACGCTGGCTATGTCA | E69-R |  | GAACGCTTCGAAAAGTGGAG |
| E19-F | (CCG)5 | ACGCACATTCCTGCTGTTC | E70-F | (GCAG)5 | GAACACCGTATAAGCCGCA |
| E19-R |  | CTGGTTCTCAGCAAGCCTCT | E70-R |  | GATGTCCATGCCTACGACG |
| E20-F | (CTG)5 | GGCTGTTGCTGTTGTTGTTG | E71-F | (GGAG)5 | ATGACGGTGAAACACAACGA |
| E20-R |  | CATGATGCAGGACTACGCC | E71-R |  | GCTCAGACTAGAGTGCGCGT |
| E21-F | (CTG)6 | GTGGTCCTGTGATGCCCTAC | E72-F | (GGAC)5 | CCGCAGCTATCTCGTCTTTG |
| E21-R |  | CCCAGCAAACTGTCCAAAAG | E72-R |  | GAGACCGGAAAAGGAAAAGG |
| E22-F | (A)10 | GAAGCAAAAGGGGCTGCTAT | E73-F | (TAAG)5 | CATCTTGTCCTCACGTCCAA |
| E22-R |  | TCTGCGTAGCGCGTATTATG | E73-R |  | CCGTCGAGACTTTGTTCGTT |
| E23-F | (T)18 | CGTCGCTCTTCCTTTCATCT | E74-F | (CTGA)5 | ATATCGTTAACGCACCGCCT |
| E23-R |  | GCTACAGAGTCCCAGCGAAC | E74-R |  | ACTGACTGAATGAATGGCCC |
| E24-F | (T)14 | AACTGATCAACGCTGCTCCT | E75-F | (GGTA)5 | TAGAGCGACGAGGAGGACAT |
| E24-R |  | CGAAAGCGTCGGTAGAAGAC | E75-R |  | AAATATCGACAGGAACGCGA |
| E25-F | (G)11 | TCCTAGACATCACGGGGAAC | E76-F | (CCAG)5 | AACATTGGTTCGTCGTCTCC |
| E25-R |  | ATTCTGCCAGTTTCAATGCC | E76-R |  | GTATTCGCGCTCGAGGTATC |
| E26-F | (T)10 | TAGAGAATACAATGGGGCCG | E77-F | (CAGC)5 | GGACAACACGAAAGCAGTGA |
| E26-R |  | GTCGCACATGTACTGAAGTCTC | E77-R |  | GATTTGCAGTTGCGAGGTG |
| E27-F | (T)12 | CCATAGTTGACGTGCCATTG | E78-F | (TCAG)10 | ACAACCCTTCATATTCCCCC |
| E27-R |  | CGAGCAACATGAGATGCTGT | E78-R |  | ACGCTAGCAAGCCTTCGTAA |
| E28-F | (A)10 | AATTAAGCCCTGTGCCTACG | E79-F | (CCTA)5 | GAATCTACCTCCCTCCCAGC |
| E28-R |  | GTCGTTGTTGCTGTTGCTGT | E79-R |  | ACAATTCAGGTAGTTGGCGG |
| E29-F | (T)10 | TGTCTGGATCAAAGTCTGCG | E80-F | (TTCT)5 | CTTGTCTCCGGTTTTGGAAG |
| E29-R |  | TTCCCCTCACATCATCATCA | E80-R |  | TAACAAATCGGGCCTGAAAC |
| E30-F | (C)12 | GCCAGTGCCAGATTCAGATT | E81-F | (GTAG)5 | TTGCTTGTGGGAGAAAAACC |
| E30-R |  | GCAACGAGACACTGGACAGA | E81-R |  | AAGAAGGCCCATTGTTTCCT |
| E31-F | (C)11 | TCAATCGACTTCTCAGCGTG | E82-F | (CATT)5 | GTTCACCGTTCACCGTTCTT |
| E31-R |  | AGCGAGTGCTAACGAGGAAG | E82-R |  | TGCGGTTTATGTGGATTGTG |
| E32-F | (A)10 | CTTGGACATCTTTATCCCGC | E83-F | (GCTC)5 | CGCTGTTTGGTTGCTCACTA |
| E32-R |  | AGCCTCTTCAACGTCTTCCA | E83-R |  | ACACATGCCTAACCGGAGAC |
| E33-F | (T)11 | CATCCAACTTGATGCGAATG | E84-F | (GTCA)5 | GTATCGGGAGCTCGATGGTA |
| E33-R |  | ATAGACAAACATCGGCGACC | E84-R |  | AAAGGGAATCGACGACTGTG |
| E34-F | (A)10 | CCGAGGAGATCGTAAGCAAG | E85-F | (TGGC)6 | GCGAGGATCGTCGATATGAT |
| E34-R |  | CGAGTAGAGGGTTCGTCGAG | E85-R |  | GTAGGAGGAGTGAGGGGAGC |
| E35-F | (T)10 | CCTATCCGCACGTACCACTT | E86-F | (CACCT)5 | AGACCCCGAGTTCGAATACA |
| E35-R |  | GATCTGCATGGCATTCCTTT | E86-R |  | GATCTACTGGTGCCTGTGGG |
| E36-F | (A)14 | GACGACCGGTAGCACAAGAT | E87-F | (AAGGA)5 | GATCCCCTACTCCCAACTCC |
| E36-R |  | CAACCATGCCAAACATACCA | E87-R |  | GCAAAACAAAGGCAAAAACC |
| E37-F | (C)11 | GGCTTCTGCTTGACATAGGC | E88-F | (GGATG)5 | GGTAACCCGGCTGACATTTA |
| E37-R |  | CTTGCTCCTTTTCAAGTCGC | E88-R |  | AATGAGATCCCGGAACTGTG |
| E38-F | (C)11 | TACCTGTCAGAATCGGACCC | E89-F | (GTCTC)5 | CCGAGATTGAAAAGCGCTAC |
| E38-R |  | TGACGCAAAGGTATGAGCAG | E89-R |  | CATCACATAAAGAGAATATACGAGGG |
| E39-F | (A)10 | TGTGACGCTGTAGTTCCGTC | E90-F | (CGAGA)6 | CGGGTTGGGTTATTGTATGG |
| E39-R |  | CTAGCGAGCGTACGAGAGGT | E90-R |  | TCCCAGAGGACCAAAAACAG |
| E40-F | (G)11 | TGCTCAGTGCTTATGCTTGG | E91-F | (CTCGT)5 | GCATTCCAAGTTCAGCAACA |
| E40-R |  | AACCATCAGGTTCAAAACGC | E91-R |  | GGTGTGGAGGATATGCAAGG |
| E41-F | (T)10 | GTTTTGCGCAGATTCAAACA | E92-F | (TCTAT)5 | TCATTCCTCATTCCTCGGTC |
| E41-R |  | AGAAGAAAACAACATGGCCG | E92-R |  | GCGGGGAAATACGTCTTGTA |
| E42-F | (A)16 | ACTCCGAAGCTGAACGACAT | E93-F | (CCATC)5 | TGTCTGCTTTGCTCTCGCTA |
| E42-R |  | AAGCTCAACTACGGAACCGA | E93-R |  | CCGGACAAAAGTTGAGTGGT |
| E43-F | (G)12 | TCAATTTCTGCGTGTATGCC | E94-F | (GAAAA)5 | ATATCGGGAATCACGACTGG |
| E43-R |  | GTCCAGACTGCACACAGAGC | E94-R |  | CTCTCCCGCTTTTCACTTTG |
| E44-F | (G)10 | CATCGAGGGTCTTCGAATGT | E95-F | (CTCTC)6 | ATCCCTATCGTGTCACTCGC |
| E44-R |  | ACAGTATCATTCCGGCACCT | E95-R |  | GAGAAGACGGCAAGATGGAG |
| E45-F | (A)11 | TCGCTTCTTGGGGCTAGTTA | E96-F | (GGGAGT)7 | GGTTGAGTTGAGGAGGTTCG |
| E45-R |  | AGACGTATCACCCATCCTCG | E96-R |  | TGCCTTCATCCACCTCTTTC |
| E46-F | (GC)6 | GGACTCCGTAAGGTCGATGA | E97-F | (GGCGAT)7 | TGAATGTTAAACGCGAGCAC |
| E46-R |  | TTGTGGCATGGAAATGAAGA | E97-R |  | ACTGAACTGCTGGGAATTGG |
| E47-F | (CA)7 | TGACACGTCGGGCAATAATA | E98-F | (TGCCAG)5 | CTCCTTCGTGTTCTTCTCCG |
| E47-R |  | TCGCTCTTTCCCGTTCTTTA | E98-R |  | GTTCCTGCGCAAGTCGCT |
| E48-F | (CA)7 | TAGATTTACGGCGACCATCC | E99-F | (CTTCGT)5 | TCCTCCTCTTCGTCTTCGTC |
| E48-R |  | CAGACTTGCATTGCCTCGT | E99-R |  | ACGAAGTGGGTGATGACGTT |
| E49-F | (CG)9 | GTACAGGACGGTGCACAGAG | E100-F | (GAACGG)5 | CACATCAAACAATTCACCGC |
| E49-R |  | CTCTGGAATCACGGCGCT | E100-R |  | ATCAGACTCGACACGCTCG |
| E50-F | (GT)9 | TCGTCTGCAGTATGCACCAT | E101-F | (TCCTTC)5 | CAAAGCTCCCCAAAATCTCA |
| E50-R |  | CACCCAGTTACCATTCCTGG | E101-R |  | CCGTTCGATTCCGATTACAT |
| E51-F | (GA)6 | AGGAGCACTCGACATCGTCT | E102-F | (TGGTGC)5 | CTTATATGGCCCCGCCTTAC |
| E51-R |  | CAAGAACGCGTTTTCAGGTC | E102-R |  | CCCATTCCTTTGAGCTTCAC |

**Table S3**

| Number | Pathway  ID | Pathway name | Unigene number |
| --- | --- | --- | --- |
| 1 | ko01230 | Metabolism，Overview，Biosynthesis of amino acids | 240 |
| 2 | ko03013 | Genetic Information Processing，Translation，RNA transport | 240 |
| 3 | ko04141 | Genetic Information Processing，Folding sorting and degradation，Protein processing in endoplasmic reticulum | 232 |
| 4 | ko03010 | Genetic Information Processing，Translation，Ribosome | 181 |
| 5 | ko04111 | Cellular Processes，Cell growth and death，Cell cycle - yeast | 181 |
| 6 | ko04011 | Environmental Information Processing，Signal transduction，MAPK signaling pathway - yeast | 180 |
| 7 | ko03040 | Genetic Information Processing，Transcription，Spliceosome | 177 |
| 8 | ko00230 | Metabolism，Nucleotide metabolism，Purine metabolism | 173 |
| 9 | ko03008 | Genetic Information Processing，Translation，Ribosome biogenesis in eukaryotes | 173 |
| 10 | ko01200 | Metabolism，Overview，Carbon metabolism | 169 |
| 11 | ko00500 | Metabolism，Carbohydrate metabolism，Starch and sucrose metabolism | 166 |
| 12 | ko00520 | Metabolism，Carbohydrate metabolism，Amino sugar and nucleotide sugar metabolism | 145 |
